# Association analysis of polymorphisms of G protein-coupled receptor 54 gene exons with reproductive traits in Jiaxing Black sows

**DOI:** 10.5713/ajas.18.0827

**Published:** 2019-01-04

**Authors:** Fen Wu, Wei Zhang, Qian-Qian Song, Hai-Hong Li, Ming-Shu Xu, Guo-Liang Liu, Jin-Zhi Zhang

**Affiliations:** 1 College of Animal Sciences, Zhejiang University, Hangzhou 310058, China; 2 Zhejiang Qinglian Food Company Limited, Jiaxing 314000, China

**Keywords:** Jiaxing Black Sows, G Protein-coupled Receptor 54 (*GPR54*), Single Nucleotide Polymorphisms, Reproductive Traits

## Abstract

**Objective:**

The aim of this study was to detect single nucleotide polymorphisms (SNP) of G protein-coupled receptor 54 (*GPR54*) gene and explore association of this candidate gene with reproductive traits in Jiaxing Black sows.

**Methods:**

Six pairs of primers of the gene were designed to amplify all exons thus sequences of which were detected by means of direct sequencing and then SNP loci were scanned. The effects of SNPs on total number of piglets born (TNB), number of piglets born alive (NBA), number of still born piglets (NSB), and litter weight at birth (LWB) of Jiaxing Black sows were analyzed.

**Results:**

Three SNP loci, including T3739C, C3878T and T6789C, were identified via comparison of sequencing and two genotypes (AB, BB) at each SNP site were observed. T3739C resulted in the change of amino acid (Leu→Pro) in corresponding protein, and C3878T resulted in synonymous mutation (Ile→Ile). Statistical results demonstrated that allele B was the preponderant allele at the three SNP loci and Genotype BB was the preponderant genotype. Meanwhile, Chi-Square test of these three SNPs indicated that all mutation sites fitted in Hardy-Weinberg equilibrium (p>0.05). For *GPR54*-T3739C locus, Jiaxing Black sows with genotype BB had 1.23 TNB and 1.28 NBA (p<0.01) that were more than those with genotype AB, respectively. Jiaxing Black sows that had the first two parities with genotype BB had additional 2.23 TNB, 2.27 NBA (p<0.01), and 1.94 LWB (p<0.05) compared to those with genotype AB, respectively. However, for other two loci, no significant difference was found between TNB, NBA, NSB, and LWB, and different genotypes of Jiaxing Black sows.

**Conclusion:**

In conclusion, the polymorphisms of *GPR54*-T3739C locus were significantly associated to TNB, NBA, and LWB and could be used as a potential genetic marker to improve reproductive function of Jiaxing black sows.

## INTRODUCTION

G protein-coupled receptor 54 (*GPR54*) gene, initially cloned from rat brain in 1999, is also referred to as kisspeptin 1 receptor (*KiSS-1R*) or *AXOR12* gene [1]. *GPR54*, also known as a member of the rhodopsin family of G protein-coupled receptors, is the endogenous receptor of kisspetins which are peptides derived from *KiSS-1* gene [2–4]. The human *GPR54* gene is mapped to chromosome 19p13.3 and consists of five exons interrupted by four introns with an open reading frame (ORF) of 1,197 bp encoding a protein of 398 amino acids [1,5]. The pig *GPR54* gene (GenBankTM accession number NC_010444.4) is mapped to chromosome 2q21–24 and has six exons and five introns.

*KiSS-1*/*GPR54* system is generally considered as the essential regulator of puberty onset and reproductive function in many species [6]. So far, there have been many studies that reported that *KiSS-1* gene could function as candidate gene for reproductive traits in animals, which revealed that this gene played an important role in animal reproduction [7–9]. Kisspeptins, encoded by *KiSS-1* gene, are the endogenous ligands for the *GPR54* and are able to directly stimulate gonadotropin-releasing hormone release (GnRH) via *GPR54* [10,11]. Central and peripheral administration of kisspeptins could stimulate GnRH-dependent luteinizing hormone (LH) and follicle-stimulating hormone (FSH) release, and activate the development of puberty and gonadotropic axis [6,12,13]. Thus, it’s meaningful to hypothesize that *GPR54* could make a difference in complicated molecular mechanism of animal prolificacy.

*GPR54* gene ubiquitously expressed, with high mRNA levels in placenta [14], pituitary, pancreas [4], and limbic brain regions including the hypothalamus, hippocampus, and periaqueductal gray etc [1,15]. More recent studies regarding *GPR54* mRNA *in situ* hybridization have concentrated solely on identifying its presence in GnRH neurons [16,17]. Herbison et al using dual-labeling experiments showed that essentially all *GPR54*-expressing cells in the rostral preoptic area of nervous system were GnRH neurons as well [15]. Navarro et al [6] found that hypothalamic expression of *GPR54* gene changed throughout the estrous cycle, and anoestrus sows had lower hypothalamic *GPR54* mRNA content than estrus cyclic sows significantly [18].

Numerous studies have identified that inactivating mutations in *GPR54* gene could result in idiopathic hypogonadotropic hypogonadism (IHH) with delaying puberty onset and influencing FSH or LH secretion in human [19–22], which resembled the phenomenon observed in *GPR54*-deficient mice [14,19]. Meanwhile, other reports showed that activating mutations in *GPR54* could give rise to central precocious puberty (CPP) in human with premature activation of GnRH releasing [23,24].

Up to now, there have been some research findings that have identified the *GPR54* gene is associated with puberty and reproductive performance in animals, such as human [19–21], sheep [18,25], monkey [11], mice [14,19], and forth on. According to the studies above, its possible to hypothesize that the polymorphisms of *GPR54* gene are related to sows’ reproductive function. However, the literatures concerning *GPR54* gene and sows’ prolificacy were limited. Jiaxing Black pig, a famous local breed in China, is known for its significant characteristics of high prolificacy, sexual precocity, high adaptation and roughage-resistance and so on. In the present study, Jiaxing Black sows were selected as test animals, then we explored single nucleotide polymorphisms (SNPs) in *GPR54* gene by sequencing and further analyzed its hereditary effects on reproductive traits including total number of piglets born (TNB), number of piglets born alive (NBA), number of still born piglets (NSB), and litter weight at birth (LWB) in three different parities population. The aim of this study was to offer efficient molecular genetic markers for improving sow prolificacy and breeding.

## MATERIALS AND METHODS

All procedures involving animals were conducted in accordance with Chinese guidelines for animal welfare and approved by the animal welfare committee of the Animal Science College, Zhejiang University.

### Sample collection and DNA extraction

A total of 128 Jiaxing Black sows (Zhejiang Qinglian Food Company Limited located in Jiaxing City, Zhejiang Province, PR China) were selected randomly as test objects, in which there are 47 primiparous sows (the first two parities) and 81 multiparous sows (later parities), and collected their ear tissue samples, along with the data on TNB, NBA, NSB, and LWB from each sow. Took 150 mg ear tissue and cut into pieces in an Eppendorf tube to extract DNA according to the instructions of the Tiangen Genoic DNA Extraction Kit. DNA concentration and optical density value were determined with the equipment Nano 2000, and then DNA concentration was tested by electrophoresis on 1% agarose gels. At last, the extracted DNA were stored at −20°C for subsequent experiments.

### Primer design and polymerase chain reaction amplification

According to the reference sequence of *GPR54* gene provided by GenBank, six pairs of primers were designed and utilized for amplification of exon1, exon2, exon3, exon4, exon5, and exon6 of this gene (Table 1). These primers were all designed by Primer 5.0 and synthesized by Hangzhou Tsingke Biotechnology Company Limited (Hangzhou, Zhejiang, China). Polymerase chain reaction (PCR) was performed in a 25 μL reaction volume which was composed of 2×Taq Master Mix 12.5 μL, forward primer 1 μL, reversed primer 1 μL, DNA template 2 μL, ddH_2_O 8.5 μL. PCR amplification conditions are listed as followed: pre-denaturation at 94°C for 5 min, followed by 30 cycles of denaturation at 94°C for 30 s, annealing at 55°C for 30 s, extension at 72°C for 1 min, finally extension at 72°C for 4 min. The PCR products were separated by electrophoresis on 1% agarose gels in parallel with a 2,000 bp DNA marker, then purified and recycled, ultimately sequenced by Hangzhou Tsingke Biotechnology Company Limited (China).

### Polymorphism scanning and genotyping

Reference sequence was selected from Genbank program on NCBI (https://www.ncbi.nlm.nih.gov/). Sequence data were edited by Chromas Application Ver.1.0.0.1. Multiple sequences alignments were performed with SeqMan program of DNASTAR software to identify SNPs. The coding DNA sequences of different exonic regions were conceptually translated to amino acid sequences using DNAMAN software (Lynnon Biosoft, San Ramon, CA, USA).

### Statistics analysis

Genotype frequency, gene frequency, polymorphism information content (PIC), population heterozygosity, and effective allele number at each SNP locus in *GPR54* gene were calculated using Excel, then χ^2^ Hardy-Weinberg balance test was performed. The general lineal model of SPSS 19.0 was employed for analysis of the effects of different parities and genotypes on reproductive traits (TNB, NBA, NSB, and LWB) of Jiaxing Black sows. The statistical model was Y_ijk_ = μ+P_i_+G_j_ +e_ijk_. Where Y_ijk_ is individual observation for traits, μ is overall population mean, P_i_ is effect of parity, G_j_ is effect of gene, e_ijk_ is random residual effect. Student’ T-test was used to compare differences among means.

## RESULTS

### Polymerase chain reaction amplification of *GPR54* gene exons

**Figure 1:**
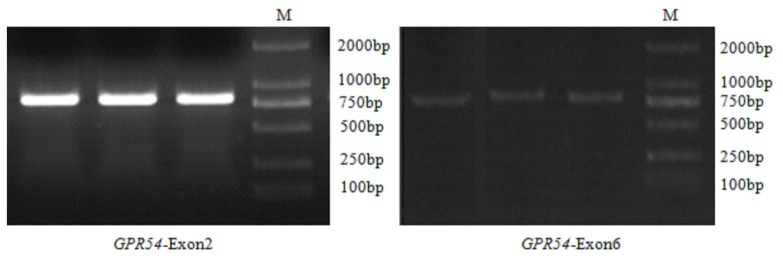
Results of GPR54-Exon2 and GPR54-Exon6 amplification. M: DL 2000 DNA marker. GPR54, G protein-coupled receptor 54.

All six exons of *GPR54* gene were successfully amplified using Jiaxing Black sows’ genetic DNA and six pairs of primers. *GPR54*-Exon2 and *GPR54*-Exon6 were selected as examples, the PCR products were detected by electrophoresis on 1% agarose gels in parallel with a 2,000 bp DNA marker. The amplified fragments sizes were approximately consistent with the target ones which were 800 bp and 786 bp in length (Figure 1), respectively, and had a good specificity so that the PCR products could be purified and recycled according to the instructions of the Gel Extraction Kit (Tsingke Biotechnology Co., Ltd, China). Splicing of exons and introns were consistent with the GT-AG rule.

### Single nucleotide polymorphisms identification and genotyping

**Figure 2:**
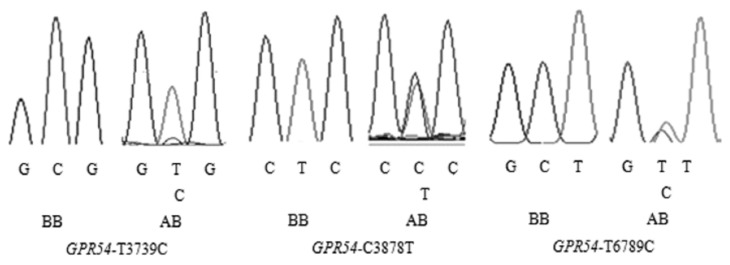
Results of polymerase chain reaction products sequencing.

Comparison of amplified sequences of Jiaxing Black sows resulted in identification of three SNPs in *GPR54* gene exons (Figure 2), in which two (T3739C and C3878T) were located within *GPR54*-Exon2 region and one (T6789C) within *GPR54*-Exon6 region. But no SNPs were found in other exonic regions of *GPR54* gene. Three SNP loci all expressed two possible genotypes (AB, BB) without genotype AA, this may reflect the small sample size. T3739C and C3878T situated in the first ORF of *GPR54* gene, the former resulted in the change of amino acid (Leu→Pro) in corresponding protein and the latter resulted in synonymous mutation (Ile→Ile). T6789C situated in untranslated regions. Thus, T3739C could be considered as a locus that might have associations with the functional traits.

Allele and genotype frequencies of different SNP loci in *GPR54* gene in Jiaxing Black sows are presented in Table 2. Allele B was the preponderant allele in all the three SNPs in *GPR54* gene and the frequencies were 0.94, 0.98, and 0.98, respectively. Genotype BB was the preponderant genotype and the frequencies were 0.88, 0.95, and 0.95, respectively. The results of Chi-square test indicated that three SNPs loci fitted in Hardy-Weinberg equilibrium (p>0.05). According to the classification of PIC, low polymorphism if PIC value <0.25, moderate polymorphism if 0.25<PIC value<0.5, and high polymorphism if PIC value >0.50, all SNP loci in *GPR54* gene were in low polymorphism in this study.

### Association analysis of single nucleotide polymorphisms with reproductive function

TNB, NBA, NSB, and LWB of Jiaxing Black sows in different parities are listed in Table 3. The TNB, NBA, NSB, and LWB in sows’ population were 12.94, 12.29, 1.44, and 13.89, respectively. According to the comparison analysis between the first two parities and later parities, sows of the later parities had 0.96 TNB, 1.03 NBA, and 0.88 NSB more than those of sows of the first two parities respectively (p<0.01). It represented that there was a certain difference in reproductive performance in sows of different parities.

For *GPR54*-T3739C locus (Table 4), TNB, NBA, and LWB of sows with two genotypes in the three groups all showed BB> AB, but NSB showed AB>BB. Analysis results identified that sows of the total parities with genotype BB had 1.23 TNB (p< 0.01) and 1.28 NBA (p<0.01) more than those with genotype AB, respectively. Sows of the first two parities with genotype BB had 2.23 TNB (p<0.01), 2.27 NBA (p<0.01), and 1.94 LWB (p<0.05) more than those with genotype AB, respectively. But for *GPR54*-C3878T (Table 5) and *GPR54*-T6789C (Table 6) loci, no significant difference was found in reproductive traits between all genotypes (p>0.05). The analysis also showed that allele B of C3878T locus had tendency to increase TNB, NBA, and LWB. These preliminary results showed an association between polymorphisms of *GPR54* gene and reproduction in Jiaxing black sows and indicated that T3739C locus had the most significant influence on reproduction in sows of the first two parities.

## DISCUSSION

A great deal of research has demonstrated that *GPR54* gene displayed abundant polymorphisms in many mammals. De Roux et al [20] amplified exon5 of *GPR54* gene from IHH patients and observed a deletion of 155 bp lying between intron4 and exon5. The sequences of the *GPR54* gene from the CPP patients revealed the presence of six SNPs including 855061G/A, 855765A/G, 856737G/A, 859955C/A, 860460G/A and 860868C/G as well [24]. Tang et al [25] reported that there were two mutations of *GPR54* gene in Small Tail Han sheep, one nucleotide transition mutation (A125G) and one 5 bp deletion/insertion (TTCTT) mutation at the 163–167 loci in 5′-regulatory region of *GPR54* gene. Chu et al [26] reported that two mutations (T2360C and A2411C) were only found in *GPR54*-Exon2 in prolific Hu sheep. A comparison of 45 amplified sequences of Indian indigenous goats resulted in identification of 2 novel SNPs, one was C1122T in exon1 and the other was T1830T in intron1 [27]. Feng et al [28] reported that five mutations, including C96T, T137C, G176A, G825A and C981T, were identified in exon1 and partial exon5 of *GPR54* gene in Jining Grey goats. Cao et al [29] showed three mutation loci in exon5 of *GPR54* gene in five goat breeds, G4014A, G4136A, and C4152T. Li et al [18] reported that seven SNPs (T245C, C384T, T1411C, A1635G, G1766T, C2488A, and T3295C) were identified in the White Duroc×Erhualian intercross pigs and the mutation T245C showed quite different allele distribution in Chinese and Western breeds. The present study also detected three SNPs (T3739C, C3878T, and T6789C) in *GPR54* gene in Jiaxing Black sows. The results all above together indicated a significant difference in the distribution of polymorphisms of *GPR54* gene in different species, which may be related to the biological evolution of the gene.

According to correlation analysis, to some extent, we assumed that the polymorphisms of *GPR54* gene were possibly related to reproductive function in Jiaxing Black sows and verified that T3739C could be a potential marker-assisted selection site that played an important role in reproduction trait. Apart from this present study, there have been many other studies indicated that *GPR54* gene could have some effects on reproduction in animals. A series of literatures had identified the loss-of-function mutations of *GPR54* gene could result in IHH in human and mice with impairment of pubertal maturation and reproductive function [14,19–21]. Teles et al [23] identified an autosomal dominant *GPR54* mutation (Arg386Pro) that led to prolonged activation of intracellular signaling pathways in response to kisspeptins and appeared to be associated with CPP. Luan et al [24] also found a nonsynonymous mutation was statistically related to some cases of CPP. In addition to investigations on human beings, many studies have been reported on various animals, but the studies on pigs are still very limited. The report by Feng showed that Jining Grey goats with genotype BB and AB had 1.07 and 0.40 lambs more than those with genotype AA, respectively, besides, genotype DD and CD had 1.80 and 0.55 lambs that were more than those with CC, which suggested there was an association between alleles B, D and high litter size [28]. And as for the 4,152 locus, Jining Grey goat with genotype TT and CT had 1.02 and 0.84 kids more than those with genotype CC respectively [29]. Small Tail Han sheep ewes with genotype CC had 0.66 or 0.49 lambs more than those with genotype DD or CD, respectively, which implied that allele C at the 163 to 167 locus in *GPR54* gene may be a potential marker for improving litter size in sheep [25]. Based on this present study, the results we observed from Jiaxing Black sows echoed the reports mentioned above that the gene could weigh somewhat on hormone metabolism and thereby affect reproductive traits.

However, there were some opposite findings that showed the polymorphisms of *GPR54* gene were not associated with reproduction traits. Chu and his colleagues had found two mutations in exon2 of ovine *GPR54* gene, but the results were not able to justify the relation of *GPR54* gene to litter size in sheep [26]. Li et al [18] also found seven SNPs in *GPR54* gene, but no significant association of *GPR54* haplotypes and haplotype pairs with age at puberty was observed in White Duroc and Erhualian pigs, implying that the mutations in *GPR54* gene were not responsible for divergent age at puberty. Since the reproductive performance of animals is controlled by multi genes, in order to verify whether the *GPR54* gene is the major gene or related gene which could affect prolificacy of sows, there is a need for further studies with more of breeds and animals to confirm the association of SNPs with reproductive traits.

## CONCLUSION

The results of the present study provide, at first, the sequences of six exons as well as three SNP loci of *GPR54* gene exons in Jiaxing Black sows. The present study preliminarily showed an association between the three SNP loci and reproductive traits which including TNB, NBA, NSB, and LWB in Jiaxing Black sows, thus indicating that the *GPR54*-T6789C locus may play an important role in reproductive function. But difference in reproductive traits for different genotypes requires additional data based on more animals to confirm the significant effect. At last, it should be efficient for researchers to make use of *GPR54* gene exons polymorphisms for maker-assisted selection to improve reproductive performance of sows.

## Figures and Tables

**Table 1:** Primer sequencing for *GPR54* gene exons

Gene	Amplification region	Forward primer (5′→3′)	Reversed primer (5′→3′)
*GPR54*	Exon1	AACGCTACCATAGCTCGGACA	TGGAAGGAAAGCCCTGTTTGT
*GPR54*	Exon2	GGAAGAGTGCCACACGGTGAATG	AGCTCTCCATGTGCCACACTCTC
*GPR54*	Exon3	ACTTCTCTGCACAAGGATGTCAGC	GCCTCCTGTCAGTCACTCTGTCCA
*GPR54*	Exon4	CCTGGGCTTCTCTTCTCCTATCC	CTTAAGCGCGTCCGAGGAGC
*GPR54*	Exon5	CATCCGGGAAATGGGCTCAAT	GGGCGACACACCAGGCACTT
*GPR54*	Exon6	GGGAAGTGCCTGGTGTGTCG	GGCAGTGTGGGAAAACTTCTATTGAT

*GPR54*, G protein-coupled receptor 54.

**Table 2:** Genotype distribution and allele frequencies of three SNPs in *GPR54* gene

Gene	SNPs	Genotype frequency			Allele frequency		χ^2^	p-value	PIC	He	Ne
	
		AA	AB	BB	A	B					
*GPR54*	T3739C	-	0.12	0.88	0.06	0.94	0.50	0.48	0.01	0.11	1.12
	C3878T	-	0.05	0.95	0.03	0.97	0.10	0.75	0.05	0.05	1.06
	T6789C	-	0.05	0.95	0.02	0.98	0.07	0.79	0.04	0.05	1.05

SNPs, single nucleotide polymorphisms; GPR54, G protein-coupled receptor 54; PIC, polymorphism information content; He, heterozygosity; Ne, effective number of alleles.

**Table 3:** Reproductive function of Jiaxing Black sows

Trait	Total parities			First two parities			Later parities		
		
	Mean±SE	Max	Min	Mean±SE	Max	Min	Mean±SE	Max	Min
TNB	12.94±0.14	16.50	6.00	12.33±0.28^B^	16.50	6.00	13.29±0.13^A^	16.00	10.60
NBA	12.29±0.15	16.50	6.00	11.64±0.31^B^	16.50	6.00	12.67±0.14^A^	15.44	10.00
NSB	1.44±0.12	7.50	0.00	0.88±0.24^B^	7.00	0.00	1.76±0.12^A^	7.50	0.00
LWB	13.89±0.21	22.30	7.80	13.42±0.37	20.60	7.80	14.17±0.26	22.30	10.77

Later parities, parties from the third; SE, standard error; TNB, total number of piglets born; NBA, number of piglets born alive; NSB, number of still born piglets; LWB, litter weight at birth.

A,B Means with different superscripts in the same row differ at p<0.01.

**Table 4:** Association between single nucleotide polymorphisms (T3739C) and reproductive traits

Parities	Genotype	TNB	NBA	NSB	LWB
Total parities	AA	-	-	-	-
	AB	11.85±0.44B	11.16±0.53B	1.48±0.46	12.96±0.70
	BB	13.08±0.14A	12.44±0.15A	1.43±0.12	14.02±0.22
First two parities	AA	-	-	-	-
	AB	10.43±0.56B	9.71±0.81B	1.14±0.99	11.65±0.84b
	BB	12.66±0.29A	11.98±0.31A	0.84±0.23	13.73±0.39a
Later parities	AA	-	-	-	-
	AB	13.09±0.17	12.42±0.28	1.77±0.22	14.11±0.97
	BB	13.32±0.14	12.70±0.15	1.76±0.13	14.18±0.27

TNB, total number of piglets born; NBA, number of piglets born alive; NSB, number of still born piglets; LWB, litter weight at birth; Later parities, parties from the third.

A,B Means with different superscripts in the same parities differ at p<0.01.

a,b Means with different superscripts in the same parities differ at p<0.05.

**Table 5:** Association between single nucleotide polymorphisms (C3878T) and reproductive traits

Parities	Genotype	TNB	NBA	NSB	LWB
Total parities	AA	-	-	-	-
	AB	12.19±0.49	11.47±0.53	1.39±0.43	12.92±0.58
	BB	12.98±0.14	12.34±0.15	1.44±0.13	13.95±0.22
First two parities	AA	-	-	-	-
	AB	11.67±1.01	11.17±1.17	0.33±0.33	13.33±1.41
	BB	12.38±0.24	11.67±0.32	0.92±0.21	13.42±0.39
Later parities	AA	-	-	-	-
	AB	12.58±0.47	11.69±0.52	2.19±0.31	12.61±0.34
	BB	13.33±0.13	12.72±0.14	1.74±0.12	14.25±0.27

TNB, total number of piglets born; NBA, number of piglets born alive; NSB, number of still born piglets; LWB, litter weight at birth; Later parities, parties from the third.

**Table 6:** Association between single nucleotide polymorphism (T6789C) and reproductive traits

Parities	Genotype	TNB	NBA	NSB	LWB
Total parities	AA	-	-	-	-
	AB	12.95±0.33	12.19±0.35	1.54±0.56	13.88±0.48
	BB	12.94±0.14	12.30±0.15	1.43±0.12	13.89±0.22
First two parities	AA	-	-	-	-
	AB	13.00±0.50	12.75±0.25	0.00±0.00	15.23±0.58
	BB	12.30±0.29	11.59±0.32	0.92±0.25	13.33±0.38
Later parities	AA	-	-	-	-
	AB	12.93±0.48	11.92±0.47	2.31±0.43	13.20±0.26
	BB	13.31±0.13	12.71±0.14	1.73±0.12	14.22±0.27

TNB, total number of piglets born; NBA, number of piglets born alive; NSB, number of still born piglets; LWB, litter weight at birth; Later parities, parties from the third.
